# Machine Learning Models to Predict 30-Day Mortality in Mechanically Ventilated Patients

**DOI:** 10.3390/jcm10102172

**Published:** 2021-05-18

**Authors:** Jong Ho Kim, Young Suk Kwon, Moon Seong Baek

**Affiliations:** 1 Department of Anaesthesiology and Pain Medicine, College of Medicine, Hallym University, Chuncheon Sacred Heart Hospital, Chuncheon 24253, Korea; poik99@hallym.or.kr; 2 Institute of New Frontier Research Team, Hallym University, Chuncheon 24253, Korea; 3 Department of Internal Medicine, Chung-Ang University Hospital, Chung-Ang University College of Medicine, Seoul 06973, Korea

**Keywords:** machine learning, mechanical ventilation, mortality, prediction

## Abstract

Previous scoring models, such as the Acute Physiologic Assessment and Chronic Health Evaluation II (APACHE II) score, do not adequately predict the mortality of patients receiving mechanical ventilation in the intensive care unit. Therefore, this study aimed to apply machine learning algorithms to improve the prediction accuracy for 30-day mortality of mechanically ventilated patients. The data of 16,940 mechanically ventilated patients were divided into the training-validation (83%, *n* = 13,988) and test (17%, *n* = 2952) sets. Machine learning algorithms including balanced random forest, light gradient boosting machine, extreme gradient boost, multilayer perceptron, and logistic regression were used. We compared the area under the receiver operating characteristic curves (AUCs) of machine learning algorithms with those of the APACHE II and ProVent score results. The extreme gradient boost model showed the highest AUC (0.79 (0.77–0.80)) for the 30-day mortality prediction, followed by the balanced random forest model (0.78 (0.76–0.80)). The AUCs of these machine learning models as achieved by APACHE II and ProVent scores were higher than 0.67 (0.65–0.69), and 0.69 (0.67–0.71)), respectively. The most important variables in developing each machine learning model were APACHE II score, Charlson comorbidity index, and norepinephrine. The machine learning models have a higher AUC than conventional scoring systems, and can thus better predict the 30-day mortality of mechanically ventilated patients.

## 1. Introduction

Acute respiratory failure is a common cause of mechanical ventilation in the intensive care unit (ICU), which results from various medical conditions, such as pneumonia, congestive heart failure, sepsis, or acute respiratory distress syndrome [1]. Although mechanical ventilation is indicated for respiratory support or airway protection, it is associated with higher mortality and morbidity [1,2,3,4,5,6]. Moreover, patients requiring prolonged mechanical ventilation have high long-term mortality, and tracheostomy is often needed to maintain mechanical ventilation. Therefore, accurate prediction of prognosis in mechanically ventilated patients in the ICU is important.

As such, several mortality prediction models for mechanically ventilated patients have been suggested [4,7,8,9,10,11]. However, there are few models that are focused on predicting hospital mortality, and most models included only patients with pneumonia or chronic obstructive lung disease. Conventional scoring systems such as Acute Physiologic Assessment and Chronic Health Evaluation II (APACHE II) or Sequential Organ Failure Assessment (SOFA) scores have been reported as a significant mortality predictor among mechanically ventilated patients [1,10,11,12,13,14]. However, the discrimination ability of these scoring systems have not been validated in large cohorts of patients with various types of respiratory failure.

Machine learning algorithms have been recently applied to predict various outcomes related to mechanical ventilation. These outcomes include prolonged ventilation or tracheostomy, need for mechanical ventilation, successful extubation, weaning from mechanical ventilation, and monitoring lung mechanics [15,16,17,18,19]. However, there are no studies yet on using machine learning models for predicting mortality in mechanically ventilated patients. Therefore, we aimed to apply machine learning algorithms to predict the mortality of mechanically ventilated patients. Further, we investigated whether the machine learning models have better predictive capability than do conventional scoring systems.

## 2. Materials and Methods

### 2.1. Data Source and Study Population

In this retrospective study, data were collected from the study cohort enrolled at five hospitals of Hallym University Medical Center, Republic of Korea. The hospitals were located in Seoul (Kangnam Sacred Heart Hospital and Hangang Sacred Heart Hospital), Gyeonggi Province (Hallym University Sacred Heart Hospital and Dongtan Sacred Heart Hospital), and Gangwon Province (Chuncheon Sacred Heart Hospital). The overall bed capacity was 3047 beds, and 2,598,544 outpatients and 835,543 inpatients were managed in 2019.

We evaluated consecutive adult patients (≥18 years old) who required mechanical ventilation in the ICU between 1 January 2010 and 31 December 2019. Among the 28,340 mechanically ventilated patients identified, 11,400 patients who underwent surgery (*n* = 7403) and had missing values (*n* = 3997) were excluded (Figure S1). Thus, 16,940 patients were included in the analysis.

The study was approved by the institutional review board of Chuncheon Sacred Hospital (No. 2020-11-008). The need for informed consent was waived owing to the retrospective nature of the study.

### 2.2. Data Collection and Definitions

Data were collected from the electronic medical records (EMRs) from each participating hospital using the clinical big data analytic solution Smart Clinical Data Warehouse based on the QlikView Elite Solution (Qlik, King of Prussia, PA, USA). The following information was collected from the time of mechanical ventilation initiation: age, sex, body mass index, time from hospitalization to ICU admission, time from hospitalization to mechanical ventilation initiation, APACHE II, ProVent score, Modified Early Warning Score (MEWS) [20], status post tracheostomy, transfer from skilled nursing facility, Charlson Comorbidity Index (CCI) and their variables [21], vital signs, continuous renal replacement therapy (CRRT), mode of mechanical ventilation, transfusion requirement (packed red blood cell, fresh frozen plasma, and platelet concentrate), use and type of vasopressors and inotropes (norepinephrine, epinephrine, dobutamine, dopamine, and vasopressin), use and type of corticosteroids (hydrocortisone, dexamethasone, and methylprednisolone), use and type of opioids (fentanyl and remifentanil), use and type of sedatives (propofol and midazolam), use and type of neuromuscular blockades (atracurium, cisatracurium, rocuronium, and vecuronium), and laboratory results with arterial blood gases. For longitudinal data such as vital signs or laboratory findings, we selected initial values taken on the day mechanical ventilation was initiated. To prevent errors in the dataset, we excluded patients with systolic blood pressure, heart rate, respiratory rate, and body temperature outside the ranges of 30–300 mmHg, 10–300 beats/min, 3–60 breaths/min, and 30–45 °C, respectively [22].

The ProVent score was calculated using five categories and their corresponding scores as follows: (1) age ≥ 65 years, 2 points; (2) age 50–64 years, 1 point; (3) platelets ≤ 100 × 109, 1 point; (4) use of vasopressors and hemodialysis, 1 point; and (5) non-trauma, 1 point [7]. The maximum score was 7. The MEWS is a bedside tool for the prediction of increased risk of clinical deterioration and uses five physiological parameters including systolic blood pressure, pulse rate, respiratory rate, temperature, and level of conscious state [20]. The allocated diagnoses for each patient were categorized using the Korean Standard Classification of Diseases-7 codes, which is a Korean version of the International Classification of Diseases-10 (ICD-10). CCI variables were categorized according to ICD-10 codes. These variables were included as features in developing the machine learning algorithms (Table S4). The primary outcome was mortality within 30 days from the initiation of mechanical ventilation in the ICU.

### 2.3. Machine Learning Algorithms

The data set involved patient variables. We divided the dataset into the training-internal validation set and external validation sets to prevent model overfitting. The test set (17%, *n* = 2952) consisted of data from Chuncheon Sacred Heart Hospital to apply the machine learning model to an independent data set. The data from other four hospitals were used for training-validation (83%, *n* = 13,988). The training-internal validation set was further divided into the training set and internal validation set at a ratio of 4:1 with the same percentage of deaths. Datasets were standardized using min–max scaling. Supervised learning is a machine learning task that learns a function and maps inputs to outputs based on example input-output pairs. All machine learning used in this study was supervised learning. We used five machine learning algorithms, namely, balanced random forest (BRF), light gradient boosting machine (LGBM), extreme gradient boost (XBG), multilayer perceptron (MLP), and logistic regression (LR) [23,24]. LR is one of the regression algorithms that predicts whether data will fall into a specific category with a continuous probability between 0 and 1. Then, based on the probability, the algorithm decides which category the specific data belongs to, and ultimately solves the classification problem. MLP is a neural network in which one or more intermediate layers exist between an input layer and an output layer. The intermediate layer between the input layer and the output layer is called a hidden layer. The network is connected in the direction of the input layer, the hidden layer, and the output layer, and there is no connection within each layer and a direct connection from the output layer to the input layer, which is a feedforward network. It differs from logistic regression in that there can be one or more nonlinear layers called hidden layers. Random forest in machine learning is a type of ensemble learning method used for classification and regression analysis and operates by outputting classification or regression analysis from a plurality of decision trees constructed in the training process. The biggest characteristic of random forest is that trees have slightly different characteristics due to randomness. This property makes the predictions of each tree uncorrelated and consequently improves the generalization performance. In addition, randomization makes the forest robust even for noise-containing data. Extreme gradient boost (XGB) is one of the gradient boosting methods. Optimized gradient boosting algorithm through parallel processing, tree-pruning, handling missing values, and regularization to void overfitting/bias. LGBM works differently from the existing gradient boosting algorithm. Existing boosting models use a method of increasing the tree level-wise, but LGBM uses leaf-wise tree division. Existing trees used level-wise partitioning to reduce the tree depth, but LGBM models behave differently, and level-wise tree analysis needs to be balanced, so the tree depth is reduced. Instead, there is a disadvantage of adding an operation to balance it. LGBM does not balance the tree and proceeds by continuously dividing the leaf nodes. Therefore, an asymmetric and deep tree is created, but when creating a lost leaf, leaf-wise has the advantage of reducing loss compared to level-wise.

Data imbalance can bias the machine-learning models and render them inaccurate. To overcome this problem, we trained the models after balancing the training dataset via the synthetic minority oversampling technique (SMOTE) to a 1:1 ratio in the XGB, LGBM, MLP, and LR models. The parameters used in SMOTE is as follows: SMOTENC (categorical_features = [n_fratures], k_neighbors = 5, n_jobs = None, sampling_strategy = ‘auto’). Then, we modeled the balanced random forest. The five models were trained using the basic hyperparameters and training sets. The model with the best performance was identified using a validation set. We performed 10-fold cross-validation on the training dataset and tuned the hyperparameters using grid search. The final model was validated using the internal validation and external validation test set. Model evaluation involved receiver operating characteristic (ROC) curve and area under the receiver operating characteristic curves (AUCs) using Anaconda (Python version 3.7, https://www.anaconda.com (accessed on 10 February 2021); Anaconda Inc., Austin, TX, USA), the XGBoost package version 0.90 (https://xgboost.readthedocs.io (accessed on 10 February 2021)), the LGBM package version 2.2.3 (https://lightgbm.readthedocs.io/en/latest/Python-Intro.html (accessed on 10 February 2021)), imbalanced-learn package version 0.5.0 (https://imbalanced-learn.readthedocs.io (accessed on 11 February 2021)), and scikit-learn 0.24.1(MLP, LR; https://scikit-learn.org/stable/index.html (accessed on 11 February 2021)).

### 2.4. Variable Importance

In BRF, XGB, and LGBM, we used the built-in function that calculates feature importance. In MLP and LR, permutation feature importance was used because there was no built-in function in the packages. Permutation feature importance provides a method to compute feature importance for any black-box estimator by measuring how score decreases when a feature is not available; the method is also known as Mean Decrease Accuracy [25,26].

### 2.5. Statistical Analyses

Descriptive analysis was performed to compare the characteristics between survivors and non-survivors. Categorical variables were presented as numbers (%) and were compared using the Pearson’s chi-squared test. Continuous variables were presented as mean ± standard deviation and were compared using the Student’s t test. The discrimination powers of APACHE II, ProVent, and MEWS were assessed according to the AUC evaluated with the ROC curve analysis. All analyses were performed using SPSS software (version 26.0; IBM Corporation, Armonk, NY, USA). Differences were considered statistically significant at *p*-values of <0.05.

## 3. Results

### 3.1. Patient Characteristics

The mean patient age was 67 years (SD ± 15), and 61.5% of the patients were male. In total, 5061 patients (29.9%) died within 30 days, and the mortality rates of internal and external data sets were 31.5% and 22.0%, respectively. Mortality rates of the four hospitals included in the internal validation set were as follows: 33.7%, 30.8%, 28.9%, and 33.7%, respectively. The baseline characteristics and laboratory values are presented in Table 1 and Table S1. Compared with the survivor group, the non-survivor group showed significantly higher age (69 ± 14 years vs. 66 ± 15 years, *p* < 0.001) and APACHE II score (26.3 ± 6.5 vs. 21.6 ± 6.8, *p* < 0.001). The rates of use of vasopressors, corticosteroids, neuromuscular blockers, and CRRT were also significantly higher in the non-survivor group. The most common type of ventilator mode was pressure control (*n* = 7269, 42.9%). The PaO2/FiO2 ratio was significantly lower in the non-survivor group (262 ± 176 vs. 207 ± 173) (Table 1). Further, other laboratory findings were also significantly different between the two groups.

The 30-day mortality of the training-validation set and test set was 31.5% and 22.00% (*p* < 0.001), respectively. Moreover, the medications and interventions received during mechanical ventilations were also significantly different (Tables S2 and S3).

### 3.2. Model Performance

**Figure 1:**
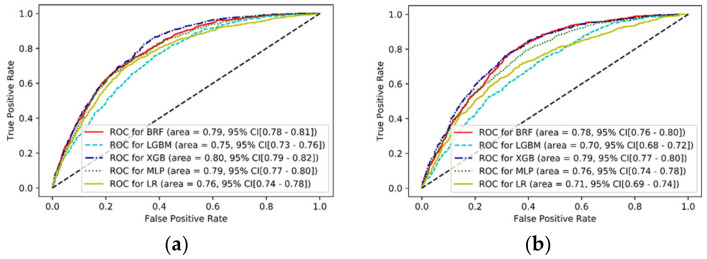
Receiver operating characteristic (ROC) curves for predicting 30-day mortality in mechanically ventilated patients: (**a**) in the internal validation, machine learning models showed AUCs from 0.75 to 0.80. (**b**) In the external validation, AUCs of XGB, BRF, MLP, LR, and LGBM were 0.79, 0.78, 0.76, 0.71, and 0.70, respectively. AUC, area under the receiver operating characteristic curve; BRF, balanced random forest; LGBM, light gradient boosting machine; XGB, extreme gradient boosting; MLP, multilayer perceptron; and LR, logistic regression.

Figure 1 demonstrates the receiver operating characteristic curves for predicting 30-day mortality in mechanically ventilated patients. In the internal validation, there was no significant difference in AUC among the BRF (0.79, 95% CI: 0.78–0.81), LGBM (0.75, 95% CI: 0.73–0.76), XGB (0.80, 95% CI: 0.79–0.82), MLP (0.79, 95% CI: 0.77–0.80), and LR (0.76, 95% CI: 0.74–0.78) models. In the test set (external validation), the discrimination functions of BRF (AUC: 0.78, 95% CI: 0.76–0.80), XGB (AUC: 0.79, 95% CI: 0.77–0.80), and MLP (AUC: 0.76, 95% CI: 0.74–0.78) were superior to those of LGBM (AUC: 0.70, 95% CI: 0.68–0.72) and LR (AUC: 0.71, 95% CI: 0.69–0.74). The other performance indicators of the algorithms are presented for each model in Table 2. BRF showed the highest sensitivity (84%), while XGB showed the highest positive predictive value (46%) as well as accuracy (76%).

**Figure 2:**
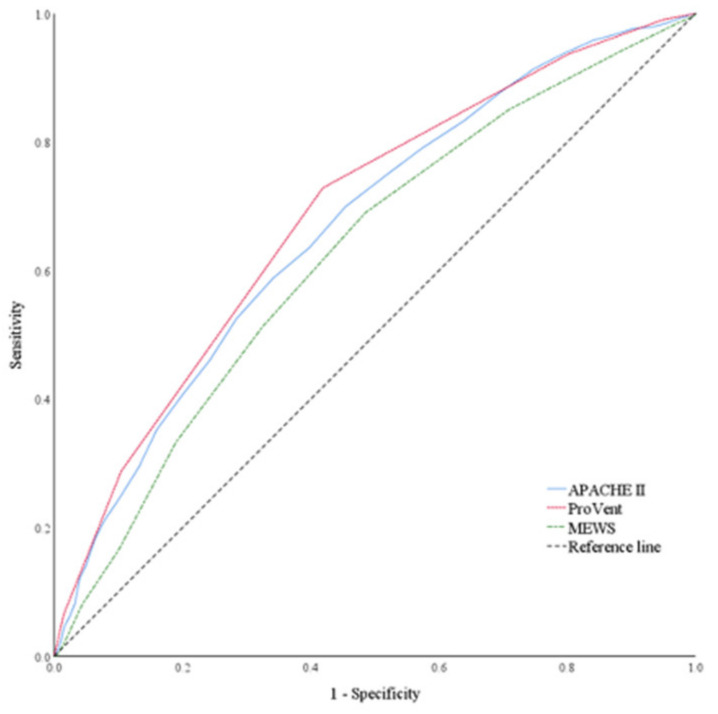
Receiver operating characteristics curves showing the performance of APACHE II (AUC: 0.67, 95% CI: 0.65–0.69), ProVent (AUC: 0.69, 95% CI: 0.67–0.71), and MEWS (AUC: 0.63, 95% CI: 0.60–0.65) for predicting 30-day mortality in mechanically ventilated patients, in the test set. APACHE, Acute Physiology and Chronic Health Evaluation; AUC, area under the curve; MEWS, Modified Early Warning Score.

In the test set, APACHE II (AUC: 0.67, 95% CI: 0.65–0.69), ProVent (AUC: 0.69, 95% CI: 0.67–0.71), and MEWS (AUC: 0.63, 95% CI: 0.60–0.65) showed lower predictive performance for 30-day mortality than did the machine learning algorithms (Figure 2). In the overall cohort, APACHE II, ProVent, and MEWS showed AUCs of 0.69 (95% CI: 0.68–0.70), 0.66 (95% CI: 0.65–0.67), and 0.67 (95% CI: 0.66–0.68), respectively (Figure S2).

### 3.3. Variable Importance

The top 10 variables in the machine learning algorithms are listed in Table 3. The most important features in the models were APACHE II in BRF and LGBM, CCI in MLP and LR, and norepinephrine in XGB. APACHE II and norepinephrine were the top predictors common across all models. Variables of ABGA including base excess and bicarbonate, and pH were considered important values in BRF. Age and comorbidities, such as chronic pulmonary disease, congestive heart failure, and diabetes were important variables in the development of the models. The results of SHapley Additive exPlanations (SHAP) of the each model were demonstrated in Supplementary Figures S3–S7.

## 4. Discussion

Few studies have evaluated the validity of predictive models of mortality in cohorts with varying types of disease conditions. This multicenter study found that machine learning models based on EMR data on the day of mechanical ventilation initiation can predict the 30-day mortality of the patients receiving mechanical ventilation in the ICU regardless of the disease condition. Although the mortality of these patients are difficult to predict, the machine learning models better predicted 30-day mortality than did the conventional scoring systems of APACHE II, ProVent, and MEWS. The BRF and XGB models showed adequate discrimination abilities (AUC, 0.78 and 0.79). The most important features in the models were APACHE II, CCI, and norepinephrine. Although APACHE II did not reveal excellent discrimination power, (AUC 0.67), the conventional scores are still useful in developing machine learning models to predict outcomes.

Machine learning models have been developed to predict mortality in patients undergoing CRRT [27], critical trauma patients [28,29], and patients in the ICU [30]. Other studies have also used these models to predict in-hospital cardiac arrest [22] and real-time mortality [31,32]. Thorsen-Meyer et al. developed a machine leaning model using a recurrent neural network in a cohort of 15,615 ICU patients with a 33% 90-day mortality [30]. The AUC at ICU admission was 0.73, and performance improved over time from AUC 0.82 after 24 h to AUC 0.85 after 72 h. Kang et al. reported that machine learning model using random forest predicted ICU mortality in 1571 patients undergoing CRRT [27]. The AUC of the machine learning model was 0.78, which was higher than those of the APACHE II and SOFA scores.

Mortality among mechanically ventilated patients are associated with factors related to patient management and complications during mechanical ventilation as well as the factors at the initiation of mechanical ventilation [1]. As such, predicting mortality in patients with mechanical ventilation can be challenging. However, our model, which used clinical data within 24 h of the mechanical ventilation initiation, showed good discrimination ability. Further, the predictive capabilities were similar to those of machine learning models described previously. In addition, unlike previous studies, the results of our study are derived from external validation in a completely independent hospital dataset.

Mechanical ventilation is one of the crucial life support devices provided in the ICU. However, mechanical ventilation is associated with markedly increased ICU costs [33]. Worldwide, there is a shortage of ICU beds because of the coronavirus disease 2019 pandemic [34]. Therefore, in terms of priority of mechanical ventilation application, rapid and accurate prediction of mortality in mechanically ventilated patients is important. Our machine learning models were trained on all ICU patients who received mechanical ventilation regardless of the disease condition. We used clinical variables that are commonly available in mechanically ventilated patients. This improved the generalizability of our machine learning models for application in the clinical setting. Furthermore, our machine learning models can predict mortality with high accuracy compared with conventional scoring systems in patients receiving mechanical ventilation. These models can be easily developed using clinical variables from electronic medical records, and therefore, can be used even in the emergency room setting though. We suggest that our machine learning models should be utilized by physicians in making clinical decisions for judging mechanical ventilation priority.

Machine learning is like a black box in that we cannot explain the processes between input and output [35]. However, it is helpful to understand variables that play a significant role in predicting performance. Although there were some discrepancies between the machine learning models, APACHE-II, norepinephrine, age, and CCI contributed significantly to the development of our machine learning models. The variables selected in each model tended to be similar to conventional studies’ results. Age, type of respiratory failure, use of inotropes, and severity scoring systems such as APACHE II and SAPS II have been considered important predictors of outcomes in patients receiving mechanical ventilation [1,12]. The models showed better discrimination abilities than the conventional scoring systems. However, APACHE II and CCI also were important factors for the development of the machine learning models. It is notable that the machine learning algorithms identified variables that were considered significant in previous studies. As machine learning for big data processing becomes more advanced, the systems for outcome prediction in critically ill patients will evolve. However, our results suggest that the conventional scoring systems will remain useful even in the big data era.

This study has some limitations. First, other variables associated with mechanical ventilation such as PEEP or plateau pressure were not included because they were not collected during the mechanical ventilation, and data were only analyzed retrospectively. Moreover, the retrospective nature of the study precluded collection of data regarding the reasons of mechanical ventilation initiation or cause of respiratory failure. Second, the AUC of SOFA score, which is one of the most used outcome prediction scores, was not presented. Third, we did not demonstrate information regarding lung heterogeneity related to the outcomes of acute respiratory distress syndrome [36]. Adding this information to the machine learning models can be beneficial to improve the prediction accuracy. In terms of the mechanical properties of the lungs, fractional order model is emerging as a tool to characterize lung function [37]. Forced oscillation technique is a non-invasive and reliable method to evaluate the lung function, and this showed a great potential in healthy, asthma, and chronic obstructive pulmonary disease patients [37,38,39]. These can be further used in machine learning algorithms for the evaluation of the respiratory systems of mechanically ventilated patients. Fourth, there is a possibility that historical bias exists due to the long duration of patient enrollment. Fifth, although the AUC of our machine learning models have shown acceptable discriminatory ability, model performance, however, needs to be improved. Therefore, further prospective studies including a large cohort with various scoring systems and variables over time are needed to improve the efficacy of machine learning models for predicting outcomes in mechanically ventilated patients. Mortality prediction in mechanically ventilated patients can be further improved if longitudinal data can be collected.

## 5. Conclusions

Compared with previous scoring models, our machine learning models of BRF, LGBM, XBG, MLP, and LR can better predict 30-day mortality in mechanically ventilated patients. Although APACHE II did not reveal excellent discrimination power, machine learning algorithms showed that the conventional scoring systems and CCI remain important factors for predicting outcomes in mechanically ventilated patients. Our findings suggest that improving discrimination power of machine learning models can help make crucial clinical decisions for patients who are less likely to benefit from mechanical ventilation.

## Figures and Tables

**Table 1:** Baseline characteristics of the patients.

Variables	Total (*n* = 16,940)	Survivors (*n* = 11,879)	Non-Survivors (*n* = 5061)	*p* Value
Age (years)	67 ± 15	66 ± 15	69 ± 14	<0.001
Male sex (%)	61.5	61.3	61.8	0.567
Interval between hospitalization and ICU admission (days)	2 ± 7	2 ± 6	3 ± 9	<0.001
Interval between hospitalization and MV initiation (days)	1 ± 6	1 ± 6	2 ± 7	<0.001
APACHE II	23 ± 4	22 ± 7	26 ± 7	<0.001
ProVent score	3 ± 1	3 ± 1	4 ± 1	<0.001
Modified early warning score	5 ± 2	4 ± 2	6 ± 2	<0.001
Transfer from skilled nursing facility (%)	9.2	8.8	10.1	0.007
Charlson comorbidity index	4 ± 3	4 ± 3	5 ± 2	0.006
Comorbidities ^a^ (%)				
Diabetes	20.5	22.2	16.4	<0.001
Congestive heart failure	18.1	19.8	14.0	<0.001
Myocardial infarction	8.5	8.8	7.8	0.037
Chronic pulmonary disease	16.5	18.4	12.1	<0.001
Liver disease	9.3	8.5	11.4	<0.001
Moderate to severe CKD	12.6	12.6	12.6	0.998
Any malignancy	20.1	19.2	22.0	<0.001
Rheumatic disease	1.6	1.4	2.2	<0.001
Dementia	7.0	7.6	5.4	<0.001
Cerebrovascular disease	26.6	27.6	24.2	<0.001
Continuous renal replacement therapy (%)	14.6	10.1	25.1	<0.001
Transfusion (%)	27.3	24.6	33.6	<0.001
Medications (%)				
Vasopressors and inotropes	50.9	44.3	66.3	<0.001
Corticosteroids	16.4	15.1	19.4	<0.001
Opioids	33.7	33.2	34.6	0.077
Sedatives	20.8	22.1	17.8	<0.001
Neuromuscular blockades	12.4	11.9	13.8	<0.001
PaO_2_/FiO_2_ ratio	246 ± 177	262 ± 176	207 ± 173	<0.001
Length of stay (day)	29 ± 36	29 ± 36	28 ± 36	0.175
ICU stay (day)	16 ± 27	16 ± 27	17 ± 26	0.767
Duration of MV (day)	11 ± 23	11 ± 23	11 ± 22	0.592

Values are presented as the mean ± SD or as %. ^a^ Comorbidities are categorized using the Charlson Comorbidity Index. ICU, intensive care unit; MV, mechanical ventilation; APACHE, Acute Physiology and Chronic Health Evaluation; CKD, chronic kidney disease; PaO_2_, partial pressure of oxygen; and FiO_2_, fraction of inspired oxygen.

**Table 2:** Performance metrics of 30-day mortality prediction models in the external validation set.

Models	AUC	Positive Predictive Value	Sensitivity	Accuracy
BRF	0.78	0.37	0.84	0.65
LGBM	0.70	0.37	0.52	0.70
XGB	0.79	0.46	0.58	0.76
MLP	0.76	0.41	0.62	0.72
LR	0.71	0.40	0.55	0.72

AUC, area under the receiver operating characteristic curve; BRF, balanced random forest; LGBM, light gradient boosting machine; XGB, extreme gradient boosting; MLP, multilayer perceptron; and LR, logistic regression.

**Table 3:** Top 10 most important variables for predicting 30-day mortality in the machine learning models.

	Machine Learning Models				
Ranking	BRF	LGBM	XGB	MLP	LR
1	APACHE II	APACHE II	Norepinephrine	CCI	CCI
2	Base excess	SpO_2_	CHF	APACHE II	APACHE II
3	HCO_3_	Respiratory rate	Chronic pulmonary disease	CHF	Age
4	Platelet	Chronic pulmonary disease	Diabetes	Chronic pulmonary disease	CHF
5	Norepinephrine	Midazolam	APACHE II	Diabetes	Chronic pulmonary disease
6	pH	CHF	SpO_2_	Norepinephrine	Diabetes
7	PaO_2_/FiO_2_	Norepinephrine	Midazolam	Age	Age group of CCI
8	Blood urea nitrogen	Age	Disease of the nervous system	Age group of CCI	Malignancy
9	eGFR	HCO_3_	Endocrine, nutritional, and metabolic disease	Transfer from skilled nursing facility	Remifentanil
10	FiO_2_	Diabetes	Mental and behavioral disorders	Malignancy	Norepinephrine

AUC, area under the receiver operating characteristic curve; BRF, balanced random forest; LGBM, light gradient boosting machine; XGB, extreme gradient boosting; MLP, multilayer perceptron; and LR, logistic regression; CCI: Charlson Comorbidity Index; CHF: congestive heart failure; eGFR: estimated glomerular filtration rate.

## Data Availability

Data sharing is not applicable to this article.

## Supplementary Materials

The following are available online at https://www.mdpi.com/article/10.3390/jcm10102172/s1, Figure S1: Flow chart of the patients, Figure S2: Receiver operating characteristics (ROC) curves of conventional scoring systems for predicting 30-day mortality in mechanically ventilated patients in total patients, Figure S3: SHAP features importance for the determinants of 30-day mortality in BRF, Figure S4: SHAP features importance for the determinants of 30-day mortality in LGBM, Figure S5: SHAP features importance for the determinants of 30-day mortality in XGB, Figure S6: SHAP features importance for the determinants of 30-day mortality in MLP, Figure S7: SHAP features importance for the determinants of 30-day mortality in LR, Table S1: Baseline characteristics and laboratory findings of the patients according to mortality, Table S2: Baseline patient characteristics according to validation dataset, Table S3: Clinical and laboratory findings according to validation dataset, Table S4: Variables included in the machine learning algorithms.
