# Supplementary material for: Machine Learning Models to Predict 30-Day Mortality in Mechanically Ventilated Patients
Source: J Clin Med. 2021 May 18;10(10):2172. doi: 10.3390/jcm10102172 (PMC8157228; doi:10.3390/jcm10102172)
Supplement: Supplementary file 1 [file jcm-10-02172-s001.zip › jcm-1174997-supplementary.pdf]

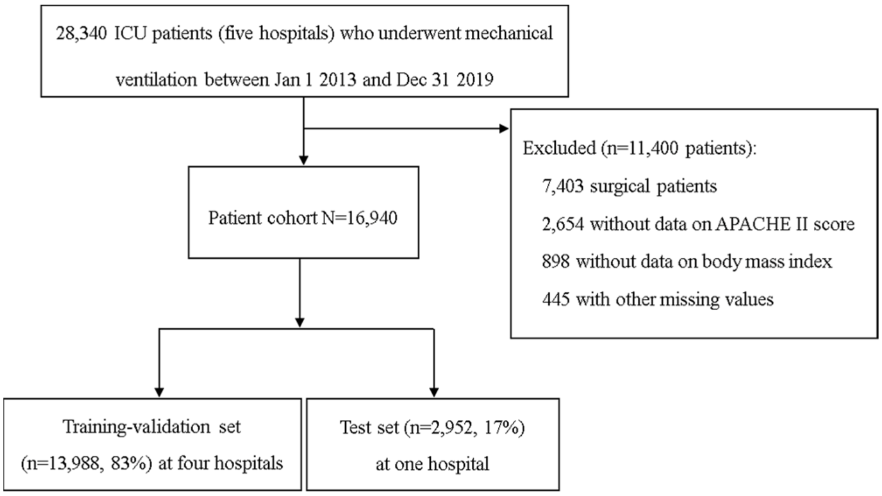

Figure S1. Flow chart of the patients.

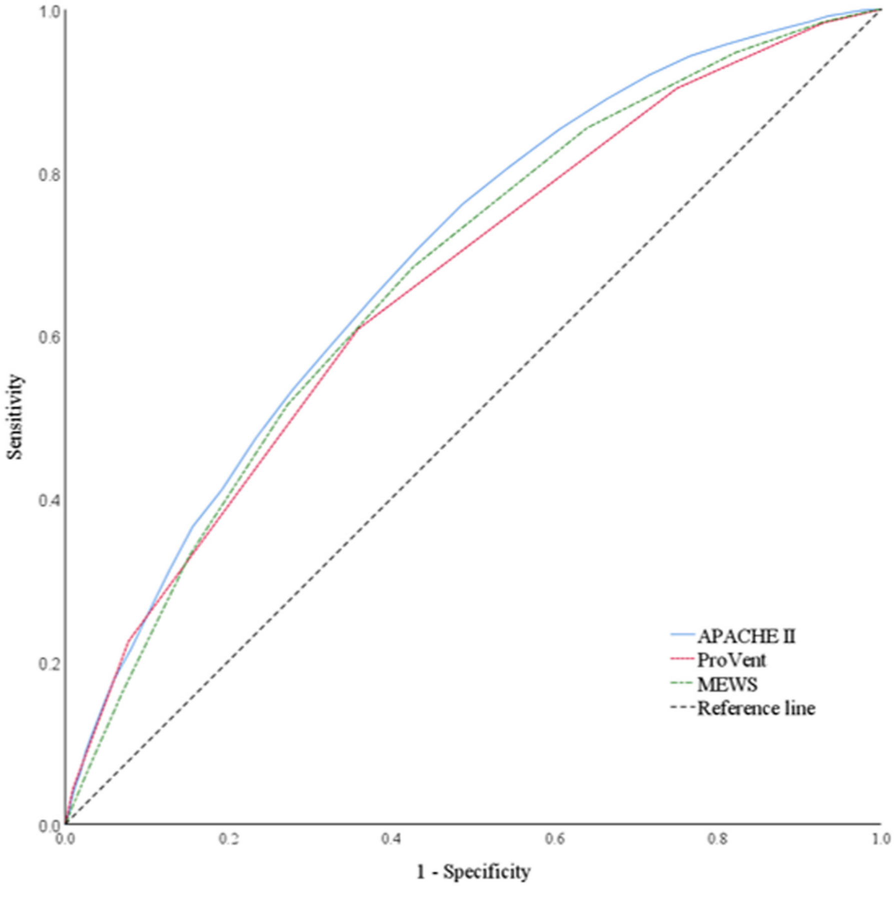

Figure S2. Receiver operating characteristics (ROC) curves of conventional scoring systems for predicting 30-day mortality in mechanically ventilated patients in total patients.

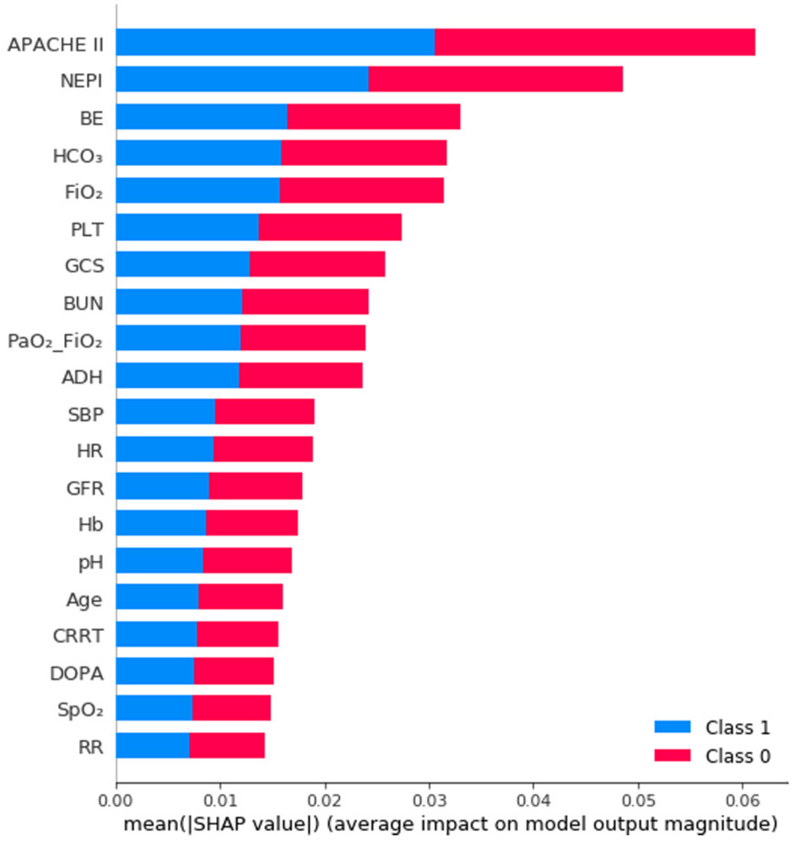

Figure S3. SHAP features importance for the determinants of 30-day mortality in BRF.

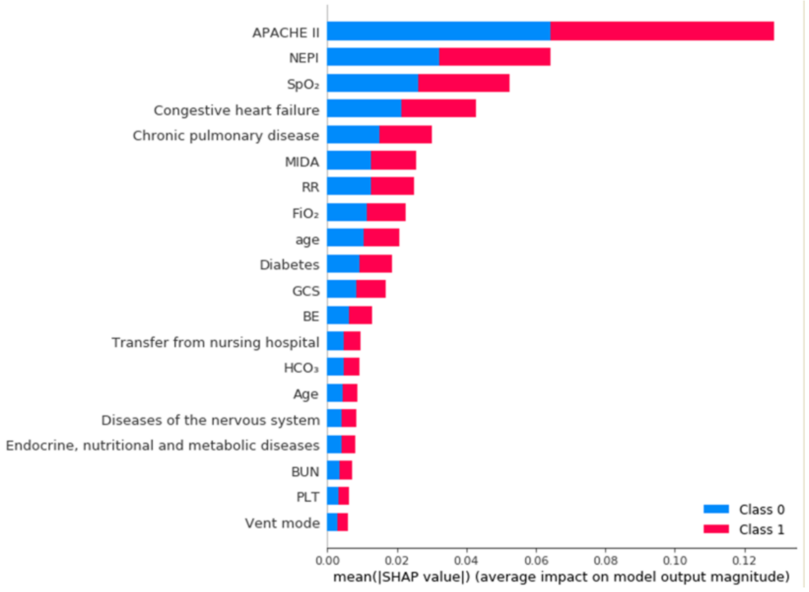

Figure S4. SHAP features importance for the determinants of 30-day mortality in LGBM.

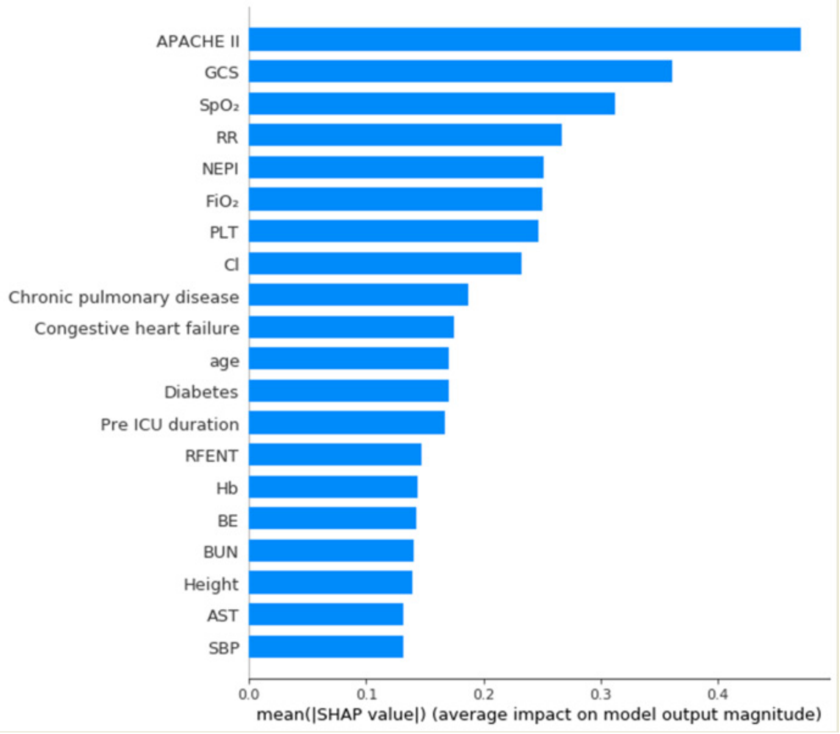

Figure S5. SHAP features importance for the determinants of 30-day mortality in XGB.

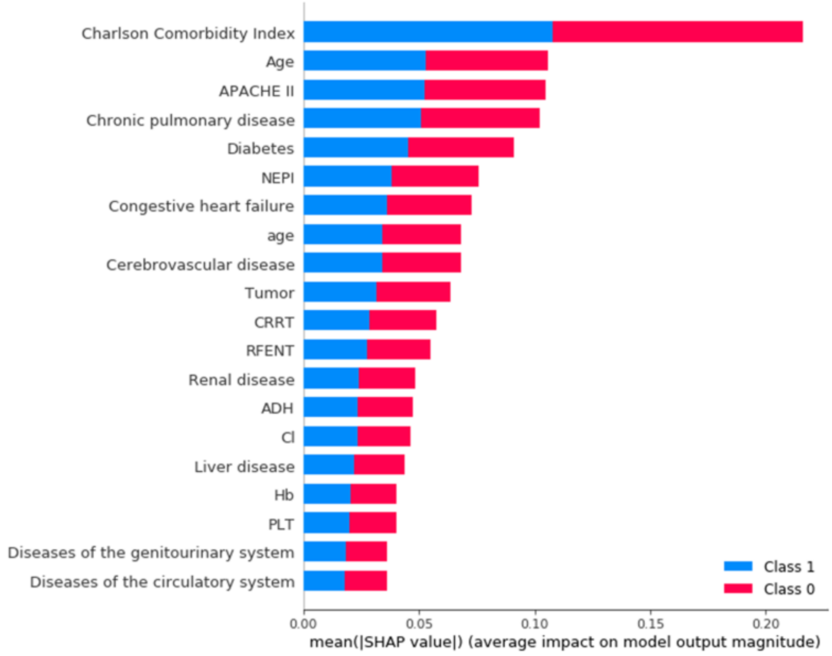

Figure S6. SHAP features importance for the determinants of 30-day mortality in MLP.

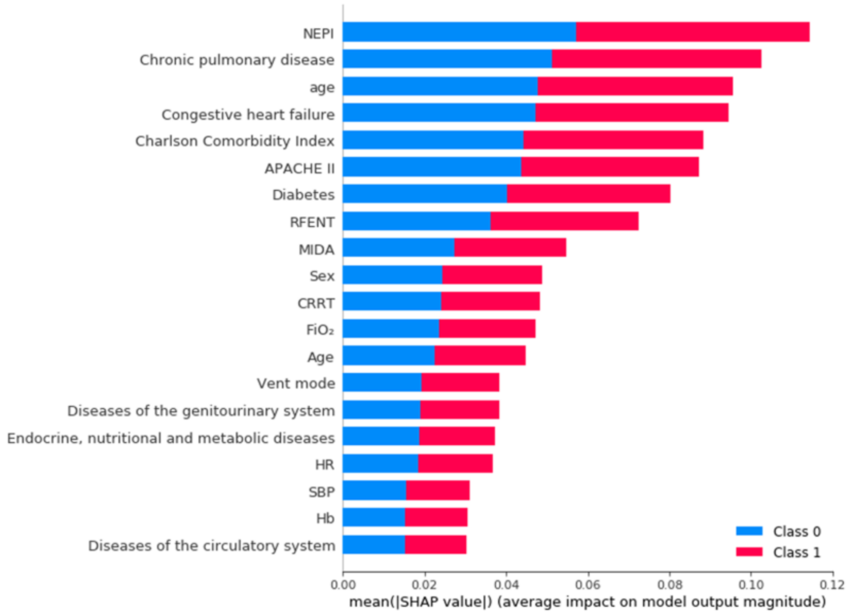

Figure S7. SHAP features importance for the determinants of 30-day mortality in LR.

Table S1. Baseline characteristics and laboratory findings of the patients according to mortality.

Variables	Total (n=16,940)	Survivors (n=11,879)	Non-survivors (n=5,061)	p Value
Age (years)	67±15	66±15	69±14	<0.001
Male sex (%)	61.5	61.3	61.8	0.567
Body mass index (kg/m^2)	22.6±4.2	22.8±4.2	22.0±4.2	<0.001
Status post tracheostomy (%)	3.6	4.3	2.1	<0.001
Diagnostic category[a] (%)				
Diseases of the respiratory system	49.0	48.9	49.4	0.552
Diseases of the circulatory system	44.3	44.6	43.4	0.151
Diseases of the digestive system	15.2	14.2	17.4	<0.001
Diseases of the genitourinary system	20.3	19.8	21.6	0.005
Certain infectious and parasitic diseases	17.0	15.9	19.5	<0.001
Endocrine, nutritional, and metabolic diseases	14.	15.0	13.3	0.004
Neoplasms	16.9	16.2	18.6	<0.001
Diseases of the nervous system	9.3	10.3	7.0	<0.001
Injury, poisoning, and certain other consequences of external causes	8.0	8.3	7.5	0.097
Diseases of the musculoskeletal system and connective tissue	4.4	4.8	3.5	<0.001
Vital signs				
Systolic blood pressure (mmHg)	121±33	124±32	112±33	<0.001
Diastolic blood pressure (mmHg)	71±22	73±21	67±23	<0.001
Mean blood pressure	88±24	90±23	82±24	<0.001
Heart rate (/min)	104±26	102±25	110±28	<0.001
Respiratory rate (/min)	17±4	16±4	18±4	<0.001
Body temperature (°C)	36.4±1.0	36.4±0.9	36.4±1.0	0.023
SpO_2 (%)	96±8	97±7	94±10	<0.001

Glasgow coma scale	7±3	7±3	6±3	<0.001
Ventilator mode (%)				<0.001
Pressure control mode	42.9	40.4	48.8	
Volume control mode	38.3	39.1	36.4	
Pressure support	6.7	7.4	5.2	
SIMV	10.4	11.3	8.5	
CPAP	1.6	1.8	1.1	
FiO2	65±23	62±22	73±24	<0.001
Arterial blood gases				
pH	7.31±0.15	7.33±0.13	7.27±0.17	<0.001
$PaCO_2$ (mmHg)	41±17	40±16	41±19	<0.001
PaO_2 (mmHg)	140±93	143±90	131±99	<0.001
HCO_3^- (mmol/L)	20±6	21±6	19±7	<0.001
BE	-5.6±7.2	-4.6±6.7	-8.0±7.7	<0.001
SaO_2 (%)	94±12	95±10	91±14	<0.001
Laboratory results				
White blood cells (×10^9/L)	13.1±8.0	12.8±6.8	13.8±10.2	<0.001
Hematocrit	34.6±7.6	35.3±7.3	32.9±8.1	<0.001
Hemoglobin (g/dL)	11.4±2.6	11.7±2.5	10.8±2.7	<0.001
Platelets (×10^9/L)	202±115	212±112	178±119	<0.001
Aspartate aminotransferase (IU/L)	191±864	139±724	314±1116	<0.001
Alanine aminotransferase (IU/L)	94±361	73±282	141±496	<0.001
Blood urea nitrogen (mg/dL)	27.4±22.0	24.9±20.2	33.4±24.7	<0.001
Serum creatinine (mg/dL)	1.6±1.7	1.5±1.8	1.9±1.7	<0.001
eGFR (mL/min/1.73m^2)	74±58	80±60	61±52	<0.001
Serum sodium (mmol/L)	139±7	139±6	138±7	<0.001
Serum potassium (mmol/L)	4.2±0.9	4.1±0.9	4.3±1.0	<0.001
Serum chloride (mmol/L)	104±7	104±7	103±8	<0.001

Values are presented as the mean±SD or as %. [a] Diagnostic categories are defined using the Korean Standard Classification of Diseases-7 (KCD-7) codes, which is a Korean version of the International Classification of Diseases-10 (ICD-10). SpO_2, pulse oximeter saturation; SIMV, synchronized intermittent mandatory ventilation; CPAP, continuous positive airway pressure; FiO_2, fraction of inspired oxygen; $PaCO_2$, partial pressure of carbon dioxide; PaO_2, partial pressure of oxygen; HCO_3^-, bicarbonate; BE, base excess; SaO_2, arterial oxygen saturation; eGFR, estimated glomerular filtration rate.

Table S2. Baseline patient characteristics according to validation dataset.

**Variables**	**Total (*n*=16,940)**	**Training-validation set (*n*=13,988)**	**Test set (*n*=2,952)**	***p* Value**
Age (years)	67±15	67±15	68±15	<0.001
Male sex (%)	61.5	61.4	61.5	0.992
Body mass index (kg/m^2)	22.6±4.2	22.6±4.2	22.7±4.2	0.186
Interval between hospitalization and ICU admission (days)	2±7	2±7	3±7	0.329
Interval between hospitalization and MV initiation (days)	1±6	1±6	2±7	<0.001
APACHE II	23±4	23±7	24±7	<0.001
ProVent score	3±1	3±1	3±1	<0.001
Modified Early Warning Score	5±2	5±2	5±2	<0.001
Status post tracheostomy (%)	3.6	3.6	3.6	0.843

Transferred from skilled nursing facility (%)	9.2	9.5	8.0	0.011
Charlson Comorbidity Index	4±3	4±2	5±3	<0.001
Comorbidities[a] (%)				
Diabetes	20.5	18.6	29.2	<0.001
Congestive heart failure	18.1	17.2	22.3	<0.001
Myocardial infarction	8.5	8.2	9.9	0.003
Chronic pulmonary disease	16.5	14.9	24.3	<0.001
Liver disease	9.3	8.0	15.6	<0.001
Moderate to severe CKD	12.6	11.9	16.2	<0.001
Any malignancy	20.1	19.6	22.1	0.002
Rheumatic disease	1.6	1.7	1.4	0.329
Dementia	7.0	5.8	12.5	<0.001
Cerebrovascular disease	26.6	25.4	32.4	<0.001
Diagnostic category[b] (%)				
Diseases of the respiratory system	49.0	49.0	49.2	0.799
Diseases of the circulatory system	44.3	42.9	50.5	<0.001
Diseases of the digestive system	15.2	14.3	19.2	<0.001
Diseases of the genitourinary system	20.3	19.8	22.9	<0.001
Certain infectious and parasitic diseases	17.0	17.4	15.0	0.002
Endocrine, nutritional, and metabolic diseases	14.4	1300	21.1	<0.001
Neoplasms	16.9	16.8	17.4	0.431
Diseases of the nervous system	9.3	9.1	10.2	0.066
Injury, poisoning, and certain other consequences of external causes	8.0	7.4	10.9	<0.001
Diseases of the musculoskeletal system and connective tissue	4.4	4.1	5.7	<0.001
Continuous renal replacement therapy (%)	14.6	14.3	16.0	0.015
Transfusion (%)	27.3	27.2	27.8	0.447
Medications (%)				
Vasopressors and inotropes	50.9	49.5	57.2	<0.001
Corticosteroids	16.4	14.6	24.7	<0.001
Opioids	33.7	31.1	45.6	<0.001
Sedatives	20.8	16.3	42.5	<0.001
Neuromuscular blockades	12.4	12.1	14.0	0.004
Length of stay (day)	29±36	28±34	31±45	0.007
ICU stay (day)	16±27	16±25	18±36	0.030
Duration of MV (day)	11±23	10±20	12±31	0.032
30-d mortality (%)	29.9	31.5	22.0	<0.001

Values are presented as the mean±SD or as %. [a] Comorbidities are categorized using the Charlson Comorbidity Index. [b] Diagnostic categories are defined using the Korean Standard Classification of Diseases-7 (KCD-7) codes, which is a Korean version of the International Classification of Diseases-10 (ICD-10) codes. ICU, intensive care unit; MV, mechanical ventilation; APACHE, Acute Physiology and Chronic Health Evaluation; CKD, chronic kidney disease.

Table S3. Clinical and laboratory findings according to validation dataset.

Variables	Total (*n*=16,940)	Training-validation set (*n*=13,988)	Test set (*n*=2,952)	*p* Value
Vital signs				
Systolic blood pressure (mmHg)	121±33	121±33	121±33	0.763
Diastolic blood pressure (mmHg)	71±22	72±22	71±22	0.070
Mean blood pressure	88±24	88±24	87±24	0.207
Heart rate (/min)	104±26	104±26	104±26	0.594
Respiratory rate (/min)	17±4	17±4	17±5	<0.001
Body temperature (°C)	36.4±1.0	36.4±0.9	36.5±1.1	0.137
SpO_2 (%)	96±8	96±8	96±7	0.725
Glasgow coma scale	7±3	7±3	7±3	0.497
Ventilator mode (%)				<0.001
Pressure control mode	7269 (42.9)	41.5	49.6	
Volume control mode	6488 (38.3)	41.1	24.8	
Pressure support	1135 (6.7)	6.4	8.2	
SIMV	1775 (10.4)	9.4	15.5	
CPAP	273 (1.6)	1.6	1.9	
FiO2	65±23	65±23	65±25	0.685
PaO_2/FiO_2 ratio	246±177	248±178	234±170	<0.001
Arterial blood gases				
pH	7.31±0.15	7.31±0.15	7.31±0.14	0.057
$PaCO_2$ (mmHg)	41±17	41±17	39±18	<0.001
PaO_2 (mmHg)	140±93	142±94	129±86	<0.001
HCO_3^- (mmol/L)	20±6	20±6	20±6	<0.001
BE	-5.6±7.2	-5.5±7.2	-6.4±7.1	<0.001
SaO_2 (%)	94±12	94±11	93±12	0.029
Laboratory results				
White blood cells (×10^9/L)	13.1±8.0	13.1±8.0	13.1±8.2	0.958
Hematocrit	34.6±7.6	34.6±7.6	34.6±7.5	0.960
Hemoglobin (g/dL)	11.4±2.6	11.5±2.6	11.4±2.5	0.489
Platelets (×10^9/L)	202±115	202±115	203±117	0.683
Aspartate aminotransferase (IU/L)	191±864	188±847	207±940	0.302
Alanine aminotransferase (IU/L)	94±361	93±345	98±427	0.453
Blood urea nitrogen (mg/dL)	27.4±22.0	27.3±22.1	28.1±21.6	0.088
Serum creatinine (mg/dL)	1.6±1.7	1.6±1.8	1.6±1.7	0.211
eGFR (mL/min/1.73m^2)	74±58	75±60	68±48	<0.001
Serum sodium (mmol/L)	139±7	138±6	141±7	<0.001
Serum potassium (mmol/L)	4.2±0.9	4.2±0.9	4.3±0.9	<0.001
Serum chloride (mmol/L)	104±7	104±7	104±8	0.050

Values are presented as the mean±SD or as %. SpO_2, pulse oximeter saturation; SIMV, synchronized intermittent mandatory ventilation; CPAP, continuous positive airway pressure; FiO_2, fraction of inspired oxygen; $PaCO_2$, partial pressure of carbon dioxide; PaO_2, partial pressure of oxygen; HCO_3^-, bicarbonate; BE, base excess; SaO_2, arterial oxygen saturation; eGFR, estimated glomerular filtration rate.

Table S4. Variables included in the machine learning algorithms.

Variables	Details
Age	
Sex	
Height	
Weight	
Body mass index	
Interval between hospitalization and ICU admission (days)	
Interval between hospitalization and MV initiation (days)	
Status post tracheostomy	
Transfer from skilled nursing facility	
APACHE II score	
Charlson Comorbidity Index (CCI)	
Age group	0, <50 years; 1, 50–59 years; 2, 60–69 years; 3, 70–79 years; and 4, ≥80 years
Systolic blood pressure	First value checked on the day of MV initiation in the ICU
Diastolic blood pressure	
Mean blood pressure	
Heart rate	
Body temperature	
SpO_2	
Ventilator mode	
Respiratory rate	
FiO_2	
Glasgow coma scale	
pH	
$PaCO_2$	
PaO_2	
HCO_3	
SaO_2	
Base excess	
PaO_2/FiO_2	
White blood cell	
Hemoglobin	
Hematocrit	
Platelet	
Aspartate aminotransferase	
Alanine aminotransferase	
Blood urea nitrogen	
Serum creatinine	
eGFR	
Serum sodium	
Serum potassium	
Serum chloride	
Packed red blood cell	Units of blood transfusion
Fresh frozen plasma	
Platelet concentrate	
Continuous renal replacement therapy	

Norepinephrine	
Epinephrine	
Dobutamine	
Dopamine	
Vasopressin	
Hydrocortisone	
Dexamethasone	
Methylprednisolone	
Propofol	
Midazolam	
Fentanyl	
Remifentanil	
Atracurium	
Cisatracurium	
Rocuronium	
Vecuronium	
**Diagnostic criteria**	**KCD-7 codes**
Certain infectious and parasitic diseases	A00–B99
Neoplasms	C00–D48
Diseases of the blood and blood-forming organs and certain disorders involving the immune mechanism	D50–D89
Endocrine, nutritional, and metabolic diseases	E00–E90
Mental and behavioral disorders	F00–F99
Diseases of the nervous system	G00–G99
Diseases of the eye and adnexa	H00–H59
Diseases of the ear and mastoid process	H60–H95
Diseases of the circulatory system	I00–I99
Diseases of the respiratory system	J00–J99
Diseases of the digestive system	K00–K93
Diseases of the skin and subcutaneous tissue	L00–L99
Diseases of the musculoskeletal system and connective tissue	M00–M99
Diseases of the genitourinary system	N00–N99
Pregnancy, childbirth, and the puerperium	O00–O99
Certain conditions originating in the perinatal period	P00–P96
Congenital malformations, deformations, and chromosomal abnormalities	Q00–Q99
Symptoms, signs, and abnormal clinical and laboratory findings, not elsewhere classified	R00–R99
Injury, poisoning, and certain other consequences of external causes	S00–T98
**Comorbidities**	**ICD-10 codes of CCI**
Myocardial infarction	I21.x, I22.x, I25.2
Congestive heart failure	I09.9, I11.0, I13.0, I13.2, I25.5, I42.0, I42.5–I42.9, I43.x, I50.x, P29.0
Peripheral vascular disease	I70.x, I71.x, I73.1, I73.8, I73.9, I77.1, I79.0, I79.2, K55.1, K55.8, K55.9, Z95.8, Z95.9
Cerebrovascular disease	G45.x, G46.x, H34.0, I60.x–I69.x
Dementia	F00.x–F03.x, F05.1, G30.x, G31.1

Chronic pulmonary disease	I27.8, I27.9, J40.x–J47.x, J60.x–J67.x, J68.4, J70.1, J70.3
Rheumatic disease	M05.x, M06.x, M31.5, M32.x–M34.x, M35.1, M35.3, M36.0
Peptic ulcer disease	K25.x–K28.x
Mild liver disease	B18.x, K70.0–K70.3, K70.9, K71.3–K71.5, K71.7, K73.x, K74.x, K76.0, K76.2–K76.4, K76.8, K76.9, Z94.4
Moderate or severe liver disease	I85.0, I85.9, I86.4, I98.2, K70.4, K71.1, K72.1, K72.9, K76.5, K76.6, K76.7
Diabetes without chronic complication	E10.0, E10.1, E10.6, E10.8, E10.9, E11.0, E11.1, E11.6, E11.8, E11.9, E12.0, E12.1, E12.6, E12.8, E12.9, E13.0, E13.1, E13.6, E13.8, E13.9, E14.0, E14.1, E14.6, E14.8, E14.9
Diabetes with chronic complication	E10.2–E10.5, E10.7, E11.2–E11.5, E11.7, E12.2–E12.5, E12.7, E13.2–E13.5, E13.7, E14.2–E14.5, E14.7
Hemiplegia or paraplegia	G04.1, G11.4, G80.1, G80.2, G81.x, G82.x, G83.0–G83.4, G83.9
Renal disease	I12.0, I13.1, N03.2–N03.7, N05.2–N05.7, N18.x, N19.x, N25.0, Z49.0–Z49.2, Z94.0, Z99.2
Any malignancy, including lymphoma and leukemia, except malignant neoplasm of skin	C00.x–C26.x, C30.x–C34.x, C37.x–C41.x, C43.x, C45.x–C58.x, C60.x–C76.x, C81.x–C85.x, C88.x, C90.x–C97.x
Metastatic solid tumor	C77.x–C80.x
AIDS/HIV	B20.x–B22.x, B24.x

ICU, intensive care unit; MV, mechanical ventilation; APACHE, Acute Physiology and Chronic Health Evaluation; CCI, Charlson Comorbidity Index; SpO_2, pulse oximeter saturation; FiO_2 , fraction of inspired oxygen; $PaCO_2$, partial pressure of carbon dioxide; PaO_2, partial pressure of oxygen; HCO_3^-, bicarbonate; SaO_2, arterial oxygen saturation; and eGFR, estimated glomerular filtration rate; KCD-7: Korean Standard Classification of Diseases-7; ICD-10, International Classification of Diseases-10; AIDS, acquired immune deficiency syndrome; HIV, human immunodeficiency virus.
